# A Web Resource for Designing Subunit Vaccine Against Major Pathogenic Species of Bacteria

**DOI:** 10.3389/fimmu.2018.02280

**Published:** 2018-10-02

**Authors:** Gandharva Nagpal, Salman Sadullah Usmani, Gajendra P. S. Raghava

**Affiliations:** ^1^Bioinformatics Centre, CSIR-Institute of Microbial Technology, Chandigarh, India; ^2^Centre for Bioinformatics, Computational and Systems Biology, Pathfinder Research and Training Foundation, Greater Noida, India; ^3^Center for Computational Biology, Indraprastha Institute of Information Technology, Okhla, India

**Keywords:** reverse vaccinology, vaccine designing, immunotherapeutic, epitopes, antigen, virulence factor, essential genes

## Abstract

Evolution has led to the expansion of survival strategies in pathogens including bacteria and emergence of drug resistant strains proved to be a major global threat. Vaccination is a promising strategy to protect human population. Reverse vaccinology is a more robust vaccine development approach especially with the availability of large-scale sequencing data and rapidly dropping cost of the techniques for acquiring such data from various organisms. The present study implements an immunoinformatic approach for screening the possible antigenic proteins among various pathogenic bacteria to systemically arrive at epitope-based vaccine candidates against 14 pathogenic bacteria. Thousand four hundred and fifty nine virulence factors and Five hundred and forty six products of essential genes were appraised as target proteins to predict potential epitopes with potential to stimulate different arms of the immune system. To address the self-tolerance, self-epitopes were identified by mapping on 1000 human proteome and were removed. Our analysis revealed that 21proteins from 5 bacterial species were found as virulent as well as essential to their survival, proved to be most suitable vaccine target against these species. In addition to the prediction of MHC-II binders, B cell and T cell epitopes as well as adjuvants individually from proteins of all 14 bacterial species, a stringent criteria lead us to identify 252 unique epitopes, which are predicted to be T-cell epitopes, B-cell epitopes, MHC II binders and Vaccine Adjuvants. In order to provide service to scientific community, we developed a web server VacTarBac for designing of vaccines against above species of bacteria. This platform integrates a number of tools that includes visualization tools to present antigenicity/epitopes density on an antigenic sequence. These tools will help users to identify most promiscuous vaccine candidates in a pathogenic antigen. This server VacTarBac is available from URL (http://webs.iiitd.edu.in/raghava/vactarbac/).

## Introduction

Evolution of existing bacterial pathogens and emergence of new pathogenic strains are continuously causing problems to mankind. To address the pathogenic challenges to the human health, researchers developed various vaccines and antibiotics during the twentieth century. A worldwide usage of such therapeutic strategies led to the expansion of structural as well as behavioral properties in various bacterial species. Diverse defense mechanisms were acquired by the known bacterial species that resulted in antibiotic tolerance or drug resistance, currently a major concern during administration of known antibiotics in pathogenic conditions. Lord Jim O'Neil and his team estimated that antimicrobial resistance could cause 10 million deaths a year by 2050 (1).

Vaccination has proved to be a promising strategem to protect human population from dreadful diseases like smallpox, polio, etc. Yet, vaccines are currently unavailable for many infectious diseases such as melioidosis caused by *Burkholderia pseudomallei* (2). Even among the existing vaccines, BCG which is heavily used vaccine against tuberculosis, is less effective in the immunocompromised and adults in tropical and subtropical region (3) and its efficacy is rapidly decreasing (4). Anthrax, DPT, Hib, Meningococcal vaccine, Pneumococcus heptavalent conjugate polysaccharide vaccine (PCV7), Pneumococcus 13-valent conjugate polysaccharide vaccine (PCV13) etc are generally considered effective at controlling disease symptoms. However, a major concern of recent vaccination programs tends to be the preference of vaccine escape mutants, i.e., the repertoire of immune targets in the pathogen is different from that used in the vaccine formulation (5). Antigenic variation and continuous bacterial evolution has necessitated designing of better vaccine candidates.

Virulence factors (VFs) have been explored to design vaccines against several pathogens such as Porcine enteric coronaviruses (CoVs) (6), Vibrio cholerae, enterotoxigenic *Escherichia coli* (7) etc. Bacterial virulence factors (VFs) are captivating, as they play a major role in establishment of infection to cause pathological conditions while surviving in a hostile environment (8). Virulence factors are thus defined as the gene products enabling the pathogen to enter, replicate, and persist in a host even in a small inoculum. These include cell surface proteins that facilitate bacterial attachment, cell surface carbohydrates which protect a bacterium, bacterial toxins, and hydrolytic enzymes that subsidize pathogenicity of bacterium (8).

In addition to virulence factors, genes essential for the living of the bacterial cell, have proved to be attractive drug targets in various pathogens such as the fungus *Aspergillus fumigatus* (9, 10), protozoan *Leishmania* species, (11) as well as bacteria like *Streptococcus pneumoniae, Haemophilus influenza*, and *Moraxella catarrhalis* (11). Essential genes are indispensable for cellular metabolism.

The last two decades witnessed an embracement of a more revolutionary vaccine development approach, namely the information driven “reverse vaccinology” which involves identification of vaccine candidates by genome or proteome sequence analysis as the primary step. High cost and practical limitations of feasibility and time have engendered a predilection toward computational techniques for rational reduction in the number of vaccine candidates to be experimentally tested. The epitope prediction methods have made possible searching of sequence sets as large as the whole proteomes of organisms with various restriction filters to arrive at novel vaccine candidates.

In order to facilitate the scientific community, researchers have developed numerous pipelines, databases, and tools for identifying vaccine candidates against specific types of diseases (12, 13). Recently, researchers have proposed potential vaccine candidates against diverse pathogens like *Ebola* (14), *Zika* (15), *M. tuberculosis* (16), *Shigella* (17), *Hepatitis B Virus* (18), *Helicobacter pylori* (19), *Vibrio cholera* (20), *Dengue* (21), etc. using *in silico* predictions. These predicted candidates help reduce the burden in experimental labs for the researchers, thus saving time and cost of vaccine development. Most of the above-mentioned studies only focus on a single pathogen and lacked identification of adjuvant candidates.

In this study, we have systemically arrived at epitope based vaccine candidate against major pathogenic bacterial species. Table 1 provides a summary of the major pathogenic manifestations of the bacterial species considered in the present study. Both, the virulence factors and the essential genes, have been considered to predict epitopes. It is a well-known fact that, diverse bacterial species and strains have evolved multiple mechanisms for evading the host immune responses, not only by producing likes of host inhibition pathway receptor ligands (“molecular mimicry”) but also by producing virulence factors resembling the intermediates of the host inhibitory pathways. Hence, peptides found in proteomes of the 1000 human genomes were removed while implementing the pipeline. To assist the scientific community all the potential vaccine candidates have been compiled and presented in the form of a database (http://webs.iiitd.edu.in/raghava/vactarbac/).

**Table 1:** Bacterial species considered in the study with the pathological conditions caused by them.

**S. No**.	**Bacterial Species**	**Human Disease**
1	*Bacillus subtilis*	food poisoning
2	*Burkholderia pseudomallei*	Melioidosis
3	*Campylobacter jejuni*	Campylobacteriosis
4	*Escherichia coli*	cholecystitis, bacteremia, cholangitis, urinary tract infection (UTI), and traveler's diarrhea, and other clinical infections such as neonatal meningitis and pneumonia
5	*Haemophilus influenzae*	pneumonia, bacteremia, meningitis, epiglottitis, septic arthritis, cellulitis, otitis media, and purulent pericarditis
6	*Helicobacter pylori*	ulcers in the stomach and small intestine
7	*Mycobacterium tuberculosis*	Tuberculosis
8	*Pseudomonas aeruginosa*	urinary tract infections, respiratory system infections, dermatitis, soft tissue infections, bacteremia, bone and joint infections, gastrointestinal infection
9	*Salmonella enteric*	Food poisoning
10	*Staphylococcus aureus*	pneumonia, meningitis, osteomyelitis, endocarditis, toxic shock syndrome, bacteremia, and sepsis
11	*Streptococcus agalactiae*	neonatal infection, septicemia, pneumonia, meningitis, chorioamnionitis
12	*Streptococcus pneumoniae*	pneumonia (infection of the lungs), ear infections, sinus infections, meningitis (infection of the covering around the brain and spinal cord), and bacteremia (blood stream infection)
13	*Streptococcus pyogenes*	streptococcal pharyngitis, rheumatic fever, rheumatic heart disease, and scarlet fever
14	*Vibrio cholera*	Cholera

## Materials and methods

### Raw data source

Proteins were taken as raw data to predict the potential vaccine candidates. We considered both virulence factors as well as essential genes of pathogenic bacteria.

#### Virulence factors

The Virulence Factor Database (VFDB) is a resource for housing the Virulence Factors (VFs) that enable a microorganism to establish itself on or within a host thus bestowing the pathogen with a potential to cause disease (22). VFDB provides two hierarchical non-redundant datasets, i.e., sets A and B. Set A is a core dataset, covering genes associated with experimentally verified virulence factors. Therefore, we have selected protein sequences of core dataset A for further analysis. The link http://www.mgc.ac.cn/VFs/Down/VFDB_setA_pro.fas.gz provides protein sequences of the VFDB database in FASTA format when uncompressed using the command “gunzip” on a unix command line or using the tool “Winzip” on a Windows platform.

#### Essential genes/ proteins

Database of Essential Genes (DEG) maintains currently known essential genomic elements, such as protein-coding genes and non-coding RNAs, among bacteria, archaea, and eukaryotes (22). These genes are mandatory for survival of the corresponding bacterium. DEG contains 18286 proteins encoded form genes experimentally found to be essential for the survival of the 32 bacteria. For our study, we restricted the analysis of bacterial essential genes belonging to 14 species that have also been included in VFDB, for the convenience of comparison and combining with VFDB analysis.

### Target selection for vaccine identification

The most important aspect of subunit vaccine designing is vaccine target selection. In case of data downloaded from VFDB (set A), it contains 2595 proteins (11) from 46 bacterial species. We have matched these bacterial species into DEG database that resulted into 14 common bacterial species. Therefore, our dataset contains only those bacterial species, which have at-least one virulence factor and one protein encoded by essential genes from VFDB and DEG respectively. DEG contains 9664 proteins corresponding to these pathogenic bacteria. These are the essential proteins of pathogenic bacteria. Some specific bacterial components, because of their function proved to be better possible candidate for designing subunit vaccine (20, 23–25) therefore; we have selected some specific significant proteins.

#### Membrane protein

These are the protein emerged from the membrane of various pathogenic bacteria. There are evidences that membrane proteins mediate pathogen entry and colonization and are also the primary target of adaptive immune response (24). A total of 420 membranous proteins were selected from DEG for further processing.

#### Envelope protein

These proteins are derived from the envelope of various pathogenic bacteria and are essential for their survival. These are important for attachment to host cell surface and also responsible for facilitating an immune response in the host cell (25). A total of 36 proteins were screened for further processing.

#### Secretion protein

Fifty-two secretory proteins corresponding to bacterial species of our interest were deposited in DEG and play important role in their survival. Secretory system of pathogenic bacteria has been heavily exploited for screening of new possible vaccine (26, 27). These proteins play many roles in promoting bacterial virulence, from enhancing attachment to eukaryotic cells, to scavenging resources in an environmental niche, to directly intoxicating target cells and disrupting their functions (28).

#### Repair protein

Bacterial damage repair mechanisms have broader roles encompassing responses to virulence as well as stress (29). Therefore we have also considered 38 proteins associated with the repair mechanism of various pathogenic bacteria.

### Epitope prediction pipeline

#### Generation of nona-peptides

In order to design new vaccine candidate, first we have generated nona-peptides. Nona-peptides were 9-mer sequences (9 residues continuous stretch of peptide) originated from the essential or virulent proteins selected for the study. In order to avoid redundancy, we have removed all duplicated nona-peptides.

#### Removal of self-epitopes

Self-tolerance must also be considered in vaccine designing, as body's immune system rarely acts against self-epitopes. Therefore, all the nona-peptides, which are also present in human body need to be removed. To achieve this, we mapped nona-peptides on 1000 human proteome, and removed all the nona-peptides which are 100% identical.

#### Epitope prediction

Our subsequent objective was to select peptides, which could activate human immune system, and generate memory cells. Therefore, we have used a pipeline for predicting different kinds of immune epitopes among these nona-peptides. This pipeline predicts; (i) B-cell epitopes using LBtope (30) (ii) MHC class II binders using ProPred (31) (iii) T-cell epitopes using CTLpred (32) and (iv) vaccine adjuvant using VaxinPAD (1).

#### IEDB mapping

**Figure 1:**
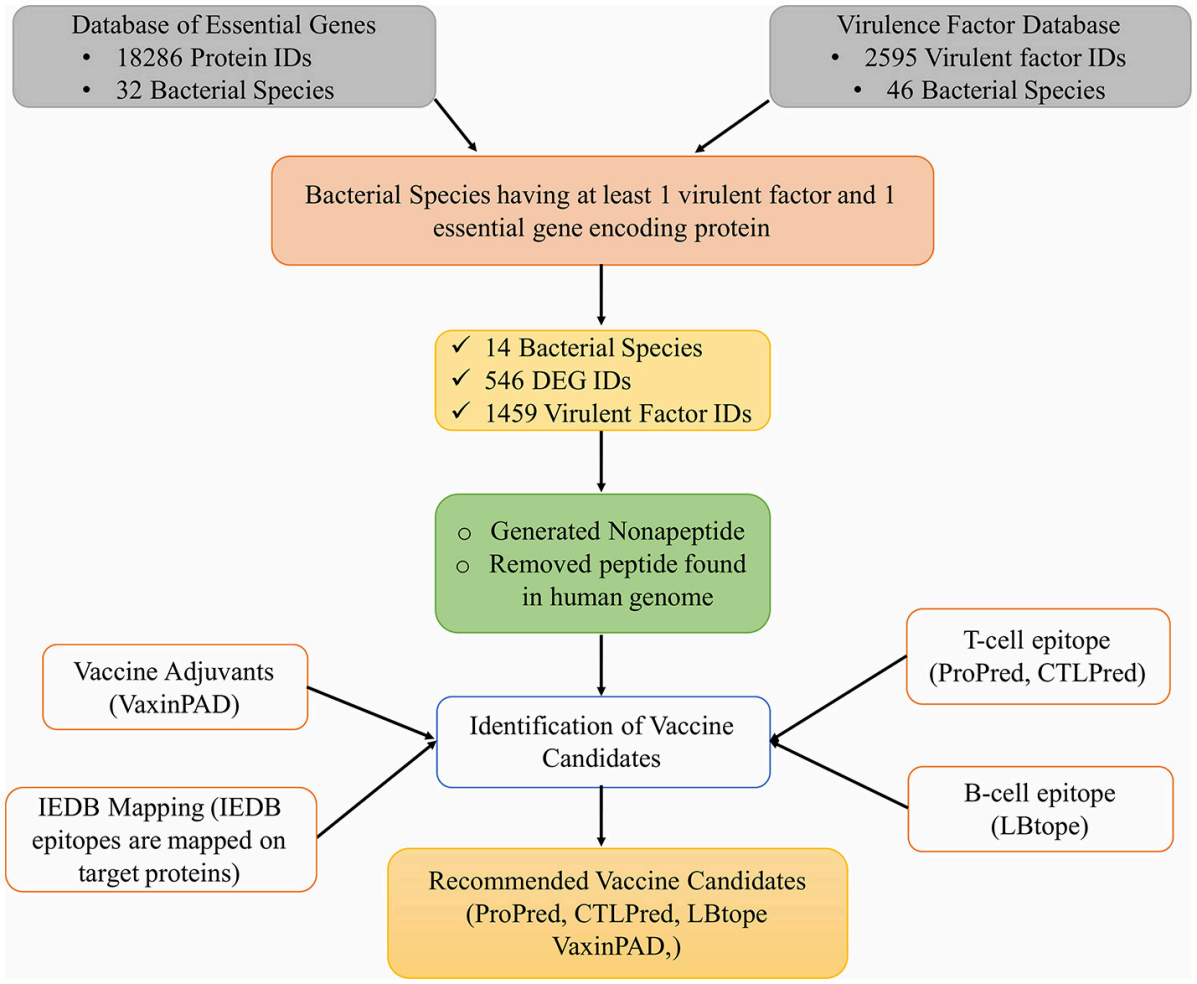
Workflow for identification of novel vaccine candidates against pathogenic bacteria.

In addition to these tools, the experimentally reported epitopes present in the Immune Epitope Database (IEDB) were also mapped on the vaccine target proteins selected for this study. Figure 1 shows the complete workflow of this study.

### Database web interface

All the predicted epitopes have been stored and presented on a web-portal named as VacTarBac. It is built on Apache HTTP server (version 2.2.17), which is installed on machine with Ubuntu as operating system. The responsive front-end, which is suitable for mobile, tablet, and desktop, was developed using HTML5, CSS3, PHP5, and JavaScript. MySQL (a relational database management system, version 5.0.51 b) was used at the back-end to manage the data.

## Results

### Screening of bacterial species

The VFDB virulence factor proteins download link is available at http://www.mgc.ac.cn/VFs/Down/VFDB_setA_pro.fas.gz, providing all the sequences in FASTA format with the headers providing the information for the protein, for example, the VFDB id, source organism, etc. From these headers, the source organism names were extracted. The DEG is available for download at http://www.essentialgene.org in the form of FASTA sequences too. Only those sequences from DEG were extracted that sourced from organism species (indicated by the headers) present in the VFDB headers too. Finally, the protein sequences from the VFDB and DEG belonged to 14 bacterial species.

### Screening of target proteins

These 14 bacterial species contain 1459 virulence factor as stated in VFDB. All these 1459 virulent factors were selected for the further study. Previous studies have advocated that bacterial proteins at specific cellular locations (20, 23–25, 33) or performing specific functions essential for cell (34) are potential candidates for designing subunit vaccine. Therefore, on the basis of function and localization with in the cell, 546 Proteins from the 14 bacterial species, were categorized as membrane, envelope, repair, and secretory proteins. Table 2 shows distribution of screened target proteins among selected 14 bacteria.

**Table 2:** Bacterial species-wise distribution of Target Proteins.

**Bacteria**	**Virulent Factors**	**Protein encoded by Essential Genes**				**Virulent Factors which are Essential Gene products**
		**Membrane Protein**	**Repair Protein**	**Secretory Protein**	**Envelope Protein**	
*Bacillus subtilis*	1	2	0	6	0	0
*Burkholderia pseudomallei*	148	27	0	4	0	0
*Campylobacter jejuni*	128	7	0	0	0	1
*Escherichia coli*	293	37	1	2	0	0
*Haemophilus influenzae*	85	10	4	0	0	0
*Helicobacter pylori*	114	8	0	0	12	1
*Mycobacterium tuberculosis*	65	241	3	23	0	14
*Pseudomonas aeruginosa*	242	18	10	8	0	2
*Salmonella enteric*	123	35	0	3	0	3
*Staphylococcus aureus*	80	11	13	6	12	0
*Streptococcus agalactiae*	45	1	0	0	0	0
*Streptococcus pneumoniae*	43	14	7	0	12	0
*Streptococcus pyogenes*	43	2	0	0	0	0
*Vibrio cholera*	49	7	0	0	0	0
Total	1459	420	38	52	36	21

The most important aspect of this rational determination of vaccine targets was to extract proteins common in both VFDB and DEG dataset. Our analysis shows that 21 IDs from VFDB dataset were identical to 35 IDs of DEG dataset. (Supplementary Table 1) These are 21 proteins from 5 bacterial species. We consider these as the most suitable vaccine targets, being both, virulence factors as well as gene products essential for survival.

### Epitope predictions

After generating the nona-peptides, redundant nona-peptides as well as self-antigens were removed by mapping on 1000 human proteome. For rest of the peptides, we predicted the immunogenicity using our prediction pipeline. LBtope, ProPred, CTLpred, and VaxinPAD helped us identify B-cell epitopes, MHC class II binders, T-cell epitopes, and adjuvants respectively. These tools integrated and implemented as a pipeline helped identify the immunogenic regions of the individual proteins in the form of nona-mer epitopes. On the other hand, the visualization tools integrated in the platform “VacTarBac” helped envisage the stretches of lengths longer than nona-mer having high density of predicted epitopes. The user may anticipate that these regions could prove to be antigens effective in the form of vaccines. Thus, for experimental investigation of the sequences that may prove to be effective vaccines, sequences longer than 9-mers containing numerous predicted epitopes could be taken up as a better strategy for the development of vaccines.

**Figure 2:**
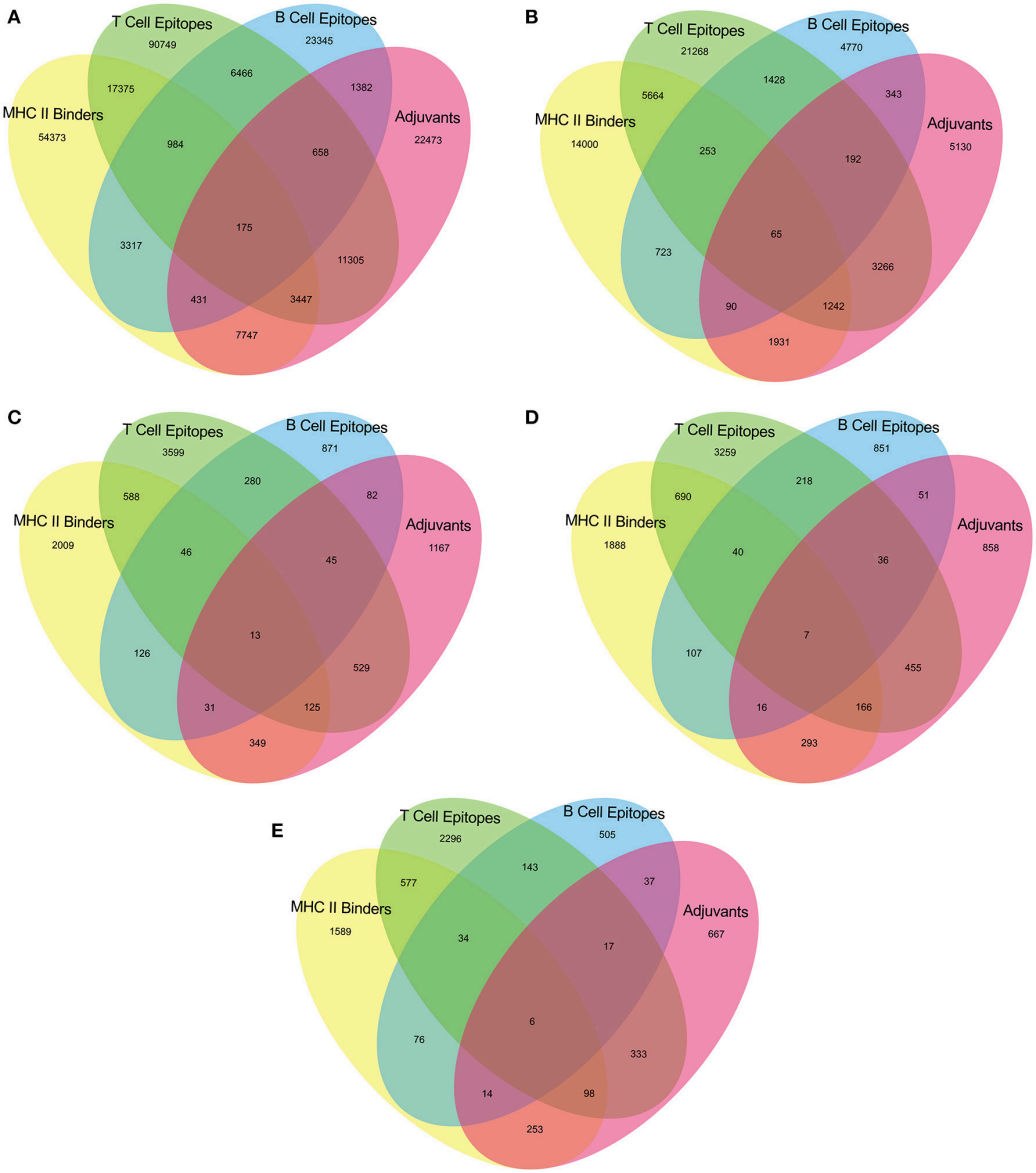
Venn diagram representing number of predicted epitopes individually as well as by intuitive approach through prediction pipeline for **(A)** Virulence factors, and essential **(B)** Membrane proteins, **(C)** Repair proteins **(D)** Secretory proteins, and **(E)** Envelope proteins.

The epitopes predicted individually by each tool of our pipeline are too many to be tested experimentally. (Table 3) Therefore, we have applied an intuitive method to arrive at a reasonable number. (Figure 2 and Supplementary Table 2) Epitopes were separately identified for T-Cell and B-Cell categories. In case of T-Cell epitopes only those were retained that were predicted T-Cell epitopes, MHC-II Binders as well as adjuvants. For B-Cell epitopes, peptides predicted to be B-Cell epitopes, MHC-II Binders and adjuvants were finalized for recommendation as therapeutics.

**Table 3:** Distribution of proteins, nona-peptides and predicted epitopes among various categories.

**Source**	**VFDB**	**Database of Essential Genes (DEG)**			
**Protein Type**	**Virulence factor**	**Membrane Protein**	**Repair Proteins**	**Secretory Protein**	**Envelope Protein**
Number of Proteins	1459	420	38	52	36
Number of Nona-peptides	1058876	482420	37823	53517	30497
Number of unique Nona-peptides	244347	60436	9866	8935	6660
Number of exclusive Nona-peptides absent in 1000 human proteome	228589	60365	9860	8935	6645
T-Cell Epitopes	131159	33378	5225	5141	3504
B-Cell Epitopes	36758	7864	1494	1326	832
MHC-II Binders	87849	23968	3287	3207	2647
Adjuvants	47618	12259	2341	1882	1425

### Epitopes assessed to be the best vaccine candidates

The aim of this study is to enhance the vaccine designing against various pathogenic bacteria by predicting potential MHC binder, B cell and T cell epitopes as well as vaccine adjuvants using a prediction pipeline. Yet, the idea of using a pipeline instead of the prediction tools individually was intended to pose stepwise filters on the numerous peptides that may be predicted as epitopes for different arms of the immune system. These filters reduce the number of epitopes to be experimentally tested and lead to a final set of peptide sequences with more possibility of activating immune system as these are recommended by all the individual tools in the prediction pipeline. Epitopes positively predicted by all the prediction tools, provided coverage of 13 out of 14 bacterial species. An organism-wise as well as vaccine target protein category-wise representation as shown in Table 4 provides more comprehensive analysis of the epitopes assessed to be the “best vaccine candidate” using all the prediction tools.

**Table 4:** Species-wise distribution of the best epitopes by filtration through all the *in silico* tools used in the study.

**Bacteria**	**Best Epitopes (T-cell Epitope**+**B-Cell Epitope**+**MHC II Binder**+**Adjuvant)**				
	**Essential Gene Proteins**				**Virulent Factors**
	**Membrane**	**Repair**	**Secretory**	**Envelope**	
*Bacillus subtilis*	0	0	0	0	0
*Burkholderia pseudomallei*	8	0	0	0	25
*Campylobacter jejuni*	0	0	0	0	17
*Escherichia coli*	7	0	1	0	42
*Haemophilus influenzae*	3	3	0	0	6
*Helicobacter pylori*	0	0	0	2	4
*Mycobacterium tuberculosis*	61	1	11	0	11
*Pseudomonas aeruginosa*	4	4	0	0	38
*Salmonella enterica*	2	0	0	0	15
*Staphylococcus aureus*	2	4	0	2	5
*Streptococcus agalactiae*	1	0	0	0	4
*Streptococcus pneumoniae*	3	1	0	2	3
*Streptococcus pyogenes*	1	0	0	0	3
*Vibrio cholerae*	0	0	0	0	6
Total number of Epitopes	92	13	12	6	179
Number of Unique Epitopes	65	13	7	6	175

*Considering all the epitopes together in this table, these belonged to 13 bacterial species out of the total 14 species considered for the study*.

### Best possible antigenic proteins

**Figure 3:**
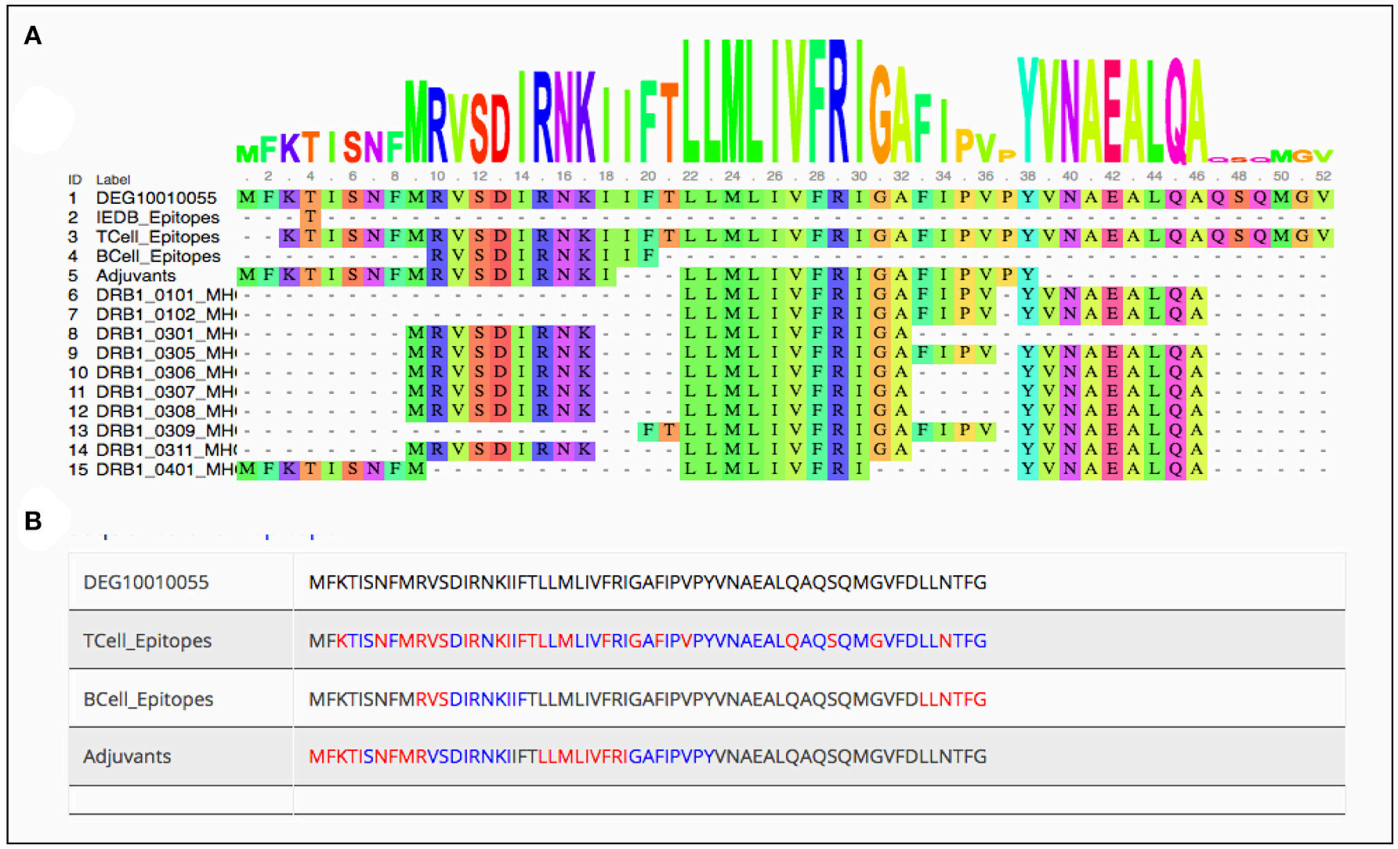
Mapping of predcited epitopes on one of the essential protein of *Bacillus subtilis* as **(A)** user friendly interactive Java-enabled view and **(B)** traditional simpler view. The blue colored sequences are the predicted 9-mer epitopes starting from red colored amino acid.

As stated earlier, the study started with 1459 virulence factor and 546 essential proteins, we have predicted epitopes from all of the generated nona-peptides. Some proteins, have high number of predicted MHC binders, T cell eptiopes, B cell epitopes as well as vaccine adjuvants. For example, the peptide syntase (pyoverdine) protein, a virulence factor of *Pseudomaonas aeruginosa* results into 8197 predcited epitopes. Such proteins or regions within the proteins could be wisely used as potential antigens to stimulate the immune system (Supplementary Table 3). Figure 3 shows the portion of an essential protein from *Bacillus subtilis*, which has a high number of predicted epitopes. This indicates that instead of experimenatlly validating a single nona-peptide, taking a longer portion of the protein, having overlapping predicted epitopes, could prove to be a better approach.

### Webserver implementation

**Figure 4:**
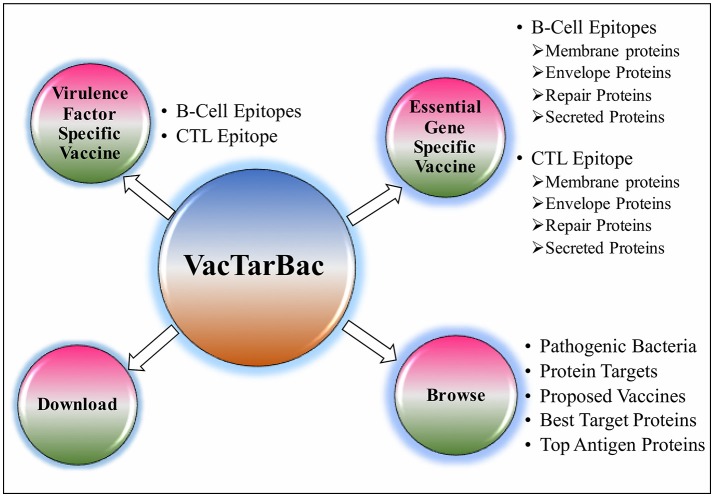
Descriptive representation of VacTarBac webserver.

To assist the scientific community in expediting the peptide-based vaccine designing against pathogenic bacteria, all the predicted potential epitopes were compiled in the form of a database and were provided as a web-based service (http://webs.iiitd.edu.in/raghava/vactarbac/). A user can browse all the recommended potential B cell and T cell epitopes designed by targeting virulence factors as well as proteins encoded by essential genes of all 14 bacterial species. Browsing by the pathogenic bacteria as well as targeted proteins considered while designing of potential epitopes is also implemented. A user can also browse the top 5 antigens from the enlisted bacteria. In addition, a list of proposed vaccine candidates is also provided in the browse option (Figure 4). For the ease of user, results have been displayed in tabular and graphical form as well as an interactive visualization.

## Discussion and conclusion

The twentieth century witnessed a remarkable success in development of antibiotics against various pathogenic bacteria but the emergence of drug resistance and toxicity caused a shift in the antimicrobial strategies toward peptide-based therapies. Several anti-bacterial peptides have been studied to check the pestiferous effects of pathogenic bacteria (35, 36). Rapid bacteriocidal activity and low propensity for resistance development are some of the major assets of these peptides, but high cost, limited stability and unknown toxicology, and pharmacokinetics are the major disadvantages (37). Contrary to antibiotics or peptide-based drugs, instigating host immune responses against the pathogen offers multifaceted management of the invasion and has been successfully achieved in the past by the use of vaccines like DPT, BCG etc against few deadly infectious diseases like tenaus, diptheria, tuberculosis etc. Consequently, active search for vaccines is underway currently for many infectious diseases caused by pathogenic bacteria, for example, melioidosis caused by *Burkholderia pseudomallei* (2). Even in case of tuberculosis, for which multiple lines of drugs and BCG vaccines are available; reduced efficacy of BCG and antibiotic resistance forced investigation of better and more potent vaccines. A recent computational effort has gone up to the strain level comparison and considered proteins as vaccine targets that were shared among tuberculoid, non-tuberculoid, and vaccine strains (16). Continuous efforts are being made by researchers to identify novel vaccine candidates against several pathogens such as *Ebola* virus (14), *Zika* virus (15), vaccinia virus (38), *Neisseria meningitidis* (39), *Corynebacterium pseudotuberculosis* (40) *Edwardsiella tarda*, and *Flavobacterium columnare*; fish pathogens (41, 42).

In past, several *in silico*, protein-based vaccine candidates have been identified, using peptide conservation score and predicted peptide-MHC binding (43) as well as molecular docking and MHC-peptide complex stabilization assay (18). In this study, we employed immunoinformatic tools to identify antigen and epitope-based vaccine candidates, having the potential to evoke one of the many arms of immune system. The present study includes proteins; virulence factors as well as proteins encoded by genes essential for survival from multiple pathogenic bacteria. The pipeline created for the identification of vaccine candidates, consists of widely used, accurate and recommended tools (12, 13). Our pipeline also includes a tool to identify peptide-based adjuvant candidates, a major advantage over other studies. The numbers of epitopes predicted by the individual tools within the pipeline that are not present in the proteome derived from the 1000 human genomes, were in thousands. For arriving at economical number of epitopes that could be tested experimentally, an intuitive rational criterion was to extract epitopes predicted positive by more than one tool in the pipeline. This would effectively mean that the selected epitope could have a predictive capability of evoking different arms of the immune system.

Upon executing the rational criteria, 1459 virulent factors from VFDB yielded 3622 nona-peptides having ability to bind with MHC-II, activate T-cell response and could act as self-adjuvants. Beside this, peptides are predicted to bind with MHC-II, activate B-cell response as well as self-adjuvants. Similarly, 420 membrane proteins from the DEG provided 1307 predicted T-cell epitopes and 155 predicted B-cell epitopes that are also MHC II binders and adjuvants. The 36 DEG envelope proteins when subjected to the epitope prediction pipeline, yield 104 predicted T-cell epitopes and 20 predicted B-cell epitopes with probability that these will bind MHC Class II molecules and would also be adjuvants. In cases of 52 secretory proteins and 38 repair proteins taken from the DEG, the predicted T-cell epitopes, and B-cell epitopes were respectively 173 and 23 for secretory proteins and 138 and 44 for the repair proteins, all of them being also the positively predicted MHC II binders as well as adjuvants.

In conclusion, the ultimate aim of the present study was the identification of epitopes capable of activating multiple wings of the human immune system arrived at by filtering the epitopes through stringent criteria. Combining the results of the VFDB and DEG datasets, this study was able to identify 252 unique epitopes predicted to be T-cell epitopes, B-cell epitopes, MHC II binders and Vaccine Adjuvants not present in the human proteome (belonging to the 1000 genomes) extracted from proteins (essential gene products and/or virulence factors) of 13 bacterial species. All the recommended vaccine candidates have been stored in a repository; VacTarBac (http://webs.iiitd.edu.in/raghava/vactarbac/) freely available on the world-wide web.

Peptides are the promising candidate as immunotherapeutic but are less immunogenic when used alone as vaccine. They need potent immunostimulatory adjuvants to effectively activate the innate and adaptive arms of immune system. In this study, we have implemented VaxinPAD in our prediction pipeline, which aids in predicting based vaccine adjuvant, thus strengthening the study. Beside this, peptide formulation is a critical task and a formulation scientist must overcome the chemical instability of peptides. The conformational fluxionality and propensity to self-associate makes peptide more difficult to formulate. Various computational tools predict the aggregation propensities of polypeptide chains such as Zyggregator (44), PASTA 2.0 (45), etc. These can be used prior to formulating the peptide-based vaccine. THPdb is a database of FDA approved protein and peptide therapeutics, and provide detailed formulation strategies of peptide-based drug available in the market (46). Although little literature exist about formulating peptide drug product but strategies used in successful peptide drugs will be helpful in designing newer formulation techniques and constituent for peptide based vaccines.

We have predicted epitopes based on highly cited, published and accurate immune epitope prediction tools. Yet, these prediction algorithms have their own limitations. Thus, the antigen or epitope should be experimentally validated before suggesting it for medical purpose. Apart from this, the platform, VacTarBac, would require continuous revamp owing to the rapidly growing sequencing data particularly that of the novel and emerging pathogenic strains. A future work for this platform may include comparison of the pathogenic proteomes at the strain level. This may lead to identification of recently acquired proteins/peptides of the pathogen that may have rendered existing therapeutic strategies against the emergent strain ineffective. Moreover, the platform, VacTarBac, still lacks information of vaccine formulations that may be added in future to help in the actual development of the vaccines. Despite such limitations, we anticipate that the current study and the current data in the repository VacTarBac, will be helpful for researchers and will boost and hasten the vaccine designing against pathogenic bacteria considered in the study.

## Availability and update of the resource

VacTarBac is freely available at http://webs.iiitd.edu.in/raghava/vactarbac/. We will update the platform at every 6 months depending upon the availability of virulence factor and essential genes information in other resources.

## Author contributions

SU and GN downloaded and processed the data. GN prepared the pipeline and predicted epitopes. SU and GN analyzed results and prepared tables and figures. SU, GN, and GR wrote the manuscript. SU developed web interface. GR conceived the idea and coordinated the project.

### Conflict of interest statement

The authors declare that the research was conducted in the absence of any commercial or financial relationships that could be construed as a potential conflict of interest.
