# Supplementary material for: A Web Resource for Designing Subunit Vaccine Against Major Pathogenic Species of Bacteria
Source: Front Immunol. 2018 Oct 2;9:2280. doi: 10.3389/fimmu.2018.02280 (PMC6190870; doi:10.3389/fimmu.2018.02280)
Supplement: Supplementary file 1 [file Table_1.DOCX]

Supplementary table 1: List of IDs representing common proteins in VFDB and DEG i.e virulence factor which are also essential proteins.

| S. No. | Virulent Factor (VFDB ID of the protein) | Essential Gene (DEG IDs of the protein) | Bacterial Species | Function | Important link |
| --- | --- | --- | --- | --- | --- |
| 1 | VFG000194 | DEG10150197 | Pseudomonas aeruginosa | putative protein in type III secretion | https://www.ncbi.nlm.nih.gov/protein/115584870/ |
| 2 | VFG000216 | DEG10150195 | Pseudomonas aeruginosa | type III export protein PscH | https://www.ncbi.nlm.nih.gov/protein/ABJ10907.1 |
| 3 | VFG000316 | DEG10080047 | Helicobacter pylori | lipopolysaccharide heptosyltransferase-1 (rfaC) | https://www.ncbi.nlm.nih.gov/protein/2313369 |
| 4 | VFG000508 | DEG10320135 | Salmonella enterica | Type three secretion system apparatus | https://www.ncbi.nlm.nih.gov/protein/CBW17437.1 |
| 5 | VFG000520 | DEG10320138 | Salmonella enterica | putative type III secretion protein | https://www.ncbi.nlm.nih.gov/protein/301157955 |
| 6 | VFG000521 | DEG10320139 | Salmonella enterica | putative type III secretion protein | https://www.ncbi.nlm.nih.gov/protein/301157956 |
| 7 | VFG001973 | DEG10310191 | Campylobacter jejuni | capsule polysaccharide export system inner membrane protein | https://www.ncbi.nlm.nih.gov/protein/CAL35554.1 |
| 8 | VFG002397 | DEG10250364,DEG10270332 | Mycobacterium tuberculosis | ESX conserved component EccB5 ESX-5 type VII secretion system protein Probable membrane protein | https://www.ncbi.nlm.nih.gov/protein/CCP44549.1 |
| 9 | VFG002398 | DEG10250366,DEG10270335 | Mycobacterium tuberculosis | ESX conserved component EccD5 ESX-5 type VII secretion system protein Probable membrane protein | https://www.ncbi.nlm.nih.gov/protein/CCP44561.1 |
| 10 | VFG002401 | DEG10270338 | Mycobacterium tuberculosis | ESX conserved component EccA5 ESX-5 type VII secretion system protein | https://www.ncbi.nlm.nih.gov/protein/CCP44564.1 |
| 11 | VFG002402 | DEG10250367,DEG10270337 | Mycobacterium tuberculosis | ESX conserved component EccE5 ESX-5 type VII secretion system protein Probable membrane protein | https://www.ncbi.nlm.nih.gov/protein/CCP44563.1 |
| 12 | VFG002403 | DEG10270336 | Mycobacterium tuberculosis | Probable proline rich membrane-anchored mycosin MycP5 (serine protease) (subtilisin-like protease) (subtilase-like) (mycosin-5) | https://www.ncbi.nlm.nih.gov/protein/CCP44562.1 |
| 13 | VFG009570 | DEG10100215 | Mycobacterium tuberculosis | Probable drugs-transport transmembrane ATP-binding protein ABC transporter | https://www.ncbi.nlm.nih.gov/protein/NP_215864.1 |
| 14 | VFG009579 | DEG10100216 | Mycobacterium tuberculosis | Probable drugs-transport transmembrane ATP-binding protein ABC transporter | https://www.ncbi.nlm.nih.gov/protein/NP_215864.1 |
| 15 | VFG022019 | DEG10250045,DEG10270049 | Mycobacterium tuberculosis | ESX conserved component EccA3 ESX-3 type VII secretion system protein | https://www.ncbi.nlm.nih.gov/protein/CCP43012.1 |
| 16 | VFG022035 | DEG10100023,DEG10250046,DEG10270050 | Mycobacterium tuberculosis | Possible conserved membrane protein | https://www.ncbi.nlm.nih.gov/protein/CCP43013.1 |
| 17 | VFG022051 | DEG10100024,DEG10250047,DEG10270051 | Mycobacterium tuberculosis | Possible conserved membrane protein | https://www.ncbi.nlm.nih.gov/protein/CCP43013.1 |
| 18 | VFG022114 | DEG10250050,DEG10270053 | Mycobacterium tuberculosis | ESX-3 secretion-associated protein EspG3 | https://www.ncbi.nlm.nih.gov/protein/BAX25924.1 |
| 19 | VFG022130 | DEG10100028,DEG10250051,DEG10270054 | Mycobacterium tuberculosis | Probable conserved transmembrane protein | https://www.ncbi.nlm.nih.gov/protein/CCP43020.1 |
| 20 | VFG022146 | DEG10100029,DEG10250052 | Mycobacterium tuberculosis | Probable membrane-anchored mycosin mycP3 (serine protease) (subtilisin-like protease) (subtilase-like) (mycosin-3) | https://www.ncbi.nlm.nih.gov/protein/CCP43021.1 |
| 21 | VFG022162 | DEG10100030,DEG10250053,DEG10270055 | Mycobacterium tuberculosis | Probable conserved transmembrane protein | https://www.ncbi.nlm.nih.gov/protein/CCP43022.1 |

Supplementary Table 2: Number of epitopes identified for various categories with potential of activating T-Cells or B-Cells along with capability of MHC-II binding as well as adjuvance.

| Protein Type | T-Cell Epitopes (+ MHC II Binders + Adjuvants) | B-Cell Epitopes (+ MHC II Binders + Adjuvants) |
| --- | --- | --- |
| Membrane protein | 1307 | 155 |
| Envelope protein | 104 | 20 |
| Secretory protein | 173 | 23 |
| Repair protein | 138 | 44 |
| Virulence factors | 3622 | 606 |

Supplementary Table 3: List of top 5 antigens having maximum number of predicted epitopes from each bacteria.

| Bacteria | Top 5 antigen ID with total number of predicted epitopes | | | | |
| --- | --- | --- | --- | --- | --- |
| Bacillus subtilis | *[DEG10010055 **(1599)](http://webs.iiitd.edu.in/raghava/vactarbac/res.php?id=DEG10010055) | [DEG10010243 (1535)](http://webs.iiitd.edu.in/raghava/vactarbac/res.php?id=DEG10010243) | [DEG10010147 (872)](http://webs.iiitd.edu.in/raghava/vactarbac/res.php?id=DEG10010147) | [DEG10010209 (826)](http://webs.iiitd.edu.in/raghava/vactarbac/res.php?id=DEG10010209) | [DEG10010123 (826)](http://webs.iiitd.edu.in/raghava/vactarbac/res.php?id=DEG10010123) |
| Burkholderia pseudomallei | *[VFG002548  (4245)](http://webs.iiitd.edu.in/raghava/vactarbac/res.php?id=VFG002548) | [VFG002461  (2360](http://webs.iiitd.edu.in/raghava/vactarbac/res.php?id=VFG002461)) | [VFG002489  (2143](http://webs.iiitd.edu.in/raghava/vactarbac/res.php?id=VFG002489)) | [VFG002522  (2096](http://webs.iiitd.edu.in/raghava/vactarbac/res.php?id=VFG002522)) | [DEG10350505  (2079](http://webs.iiitd.edu.in/raghava/vactarbac/res.php?id=DEG10350505)) |
| Campylobacter jejuni | [VFG001960  (2795)](http://webs.iiitd.edu.in/raghava/vactarbac/res.php?id=VFG001960) | [VFG001916  (2119)](http://webs.iiitd.edu.in/raghava/vactarbac/res.php?id=VFG001916) | [VFG001966  (1906)](http://webs.iiitd.edu.in/raghava/vactarbac/res.php?id=VFG001966) | [VFG043392  (1807)](http://webs.iiitd.edu.in/raghava/vactarbac/res.php?id=VFG043392) | [VFG001950  (1722)](http://webs.iiitd.edu.in/raghava/vactarbac/res.php?id=VFG001950) |
| Escherichia coli | [VFG000845  (6296)](http://webs.iiitd.edu.in/raghava/vactarbac/res.php?id=VFG000845) | [VFG001534  (5386)](http://webs.iiitd.edu.in/raghava/vactarbac/res.php?id=VFG001534) | [VFG012509  (3208)](http://webs.iiitd.edu.in/raghava/vactarbac/res.php?id=VFG012509) | [VFG000813  (2340)](http://webs.iiitd.edu.in/raghava/vactarbac/res.php?id=VFG000813) | [VFG034541  (2161)](http://webs.iiitd.edu.in/raghava/vactarbac/res.php?id=VFG034541) |
| Haemophilus influenzae | [VFG000702  (2376)](http://webs.iiitd.edu.in/raghava/vactarbac/res.php?id=VFG000702) | [DEG10050365  (2230)](http://webs.iiitd.edu.in/raghava/vactarbac/res.php?id=DEG10050365) | [VFG001154  (2126)](http://webs.iiitd.edu.in/raghava/vactarbac/res.php?id=VFG001154) | [DEG10050452  (2113)](http://webs.iiitd.edu.in/raghava/vactarbac/res.php?id=DEG10050452) | [VFG001151  (2041)](http://webs.iiitd.edu.in/raghava/vactarbac/res.php?id=VFG001151) |
| Helicobacter pylori | [DEG10080049](http://webs.iiitd.edu.in/raghava/vactarbac/res.php?id=DEG10080049)  (4570) | [VFG006577](http://webs.iiitd.edu.in/raghava/vactarbac/res.php?id=VFG006577)  (2226) | [VFG000303](http://webs.iiitd.edu.in/raghava/vactarbac/res.php?id=VFG000303)  (2202) | [VFG000279](http://webs.iiitd.edu.in/raghava/vactarbac/res.php?id=VFG000279)  (2162) | [VFG000289](http://webs.iiitd.edu.in/raghava/vactarbac/res.php?id=VFG000289)  (2034) |
| Mycobacterium tuberculosis | [DEG10270286  (3364)](http://webs.iiitd.edu.in/raghava/vactarbac/res.php?id=DEG10270286) | [DEG10250303  (3364)](http://webs.iiitd.edu.in/raghava/vactarbac/res.php?id=DEG10250303) | [DEG10270682  (3228)](http://webs.iiitd.edu.in/raghava/vactarbac/res.php?id=DEG10270682) | [DEG10250765  (3228)](http://webs.iiitd.edu.in/raghava/vactarbac/res.php?id=DEG10250765) | [DEG10270089  (3076)](http://webs.iiitd.edu.in/raghava/vactarbac/res.php?id=DEG10270089) |
| Pseudomonas aeruginosa | [VFG016041  (8197)](http://webs.iiitd.edu.in/raghava/vactarbac/res.php?id=VFG016041) | [VFG016058  (6761)](http://webs.iiitd.edu.in/raghava/vactarbac/res.php?id=VFG016058) | [VFG000161  (4779)](http://webs.iiitd.edu.in/raghava/vactarbac/res.php?id=VFG000161) | [VFG016038  (3867)](http://webs.iiitd.edu.in/raghava/vactarbac/res.php?id=VFG016038) | [VFG001231  (3189)](http://webs.iiitd.edu.in/raghava/vactarbac/res.php?id=VFG001231) |
| Salmonella enterica | [VFG002306  (2566)](http://webs.iiitd.edu.in/raghava/vactarbac/res.php?id=VFG002306) | [VFG000574  (2540)](http://webs.iiitd.edu.in/raghava/vactarbac/res.php?id=VFG000574) | [VFG000557  (2414)](http://webs.iiitd.edu.in/raghava/vactarbac/res.php?id=VFG000557) | [VFG000514  (2354)](http://webs.iiitd.edu.in/raghava/vactarbac/res.php?id=VFG000514) | [VFG000429  (1812)](http://webs.iiitd.edu.in/raghava/vactarbac/res.php?id=VFG000429) |
| Staphylococcus aureus | [DEG10170178  (3755)](http://webs.iiitd.edu.in/raghava/vactarbac/res.php?id=DEG10170178) | [VFG002409 (3196)](http://webs.iiitd.edu.in/raghava/vactarbac/res.php?id=VFG002409) | [VFG002406 (2499)](http://webs.iiitd.edu.in/raghava/vactarbac/res.php?id=VFG002406) | [DEG10020016 (2499)](http://webs.iiitd.edu.in/raghava/vactarbac/res.php?id=DEG10020016) | [VFG001305 (2304)](http://webs.iiitd.edu.in/raghava/vactarbac/res.php?id=VFG001305) |
| Streptococcus agalactiae | [VFG001339 (2326)](http://webs.iiitd.edu.in/raghava/vactarbac/res.php?id=VFG001339) | [VFG001345 (1976)](http://webs.iiitd.edu.in/raghava/vactarbac/res.php?id=VFG001345) | [VFG001348 (1770)](http://webs.iiitd.edu.in/raghava/vactarbac/res.php?id=VFG001348) | [VFG001352 (1694)](http://webs.iiitd.edu.in/raghava/vactarbac/res.php?id=VFG001352) | [VFG001334 (1536)](http://webs.iiitd.edu.in/raghava/vactarbac/res.php?id=VFG001334) |
| Streptococcus pneumoniae | [VFG001362 (2749)](http://webs.iiitd.edu.in/raghava/vactarbac/res.php?id=VFG001362) | [DEG10070047 (1591)](http://webs.iiitd.edu.in/raghava/vactarbac/res.php?id=DEG10070047) | [VFG001364 (1544)](http://webs.iiitd.edu.in/raghava/vactarbac/res.php?id=VFG001364) | [VFG001365 (1501)](http://webs.iiitd.edu.in/raghava/vactarbac/res.php?id=VFG001365) | [VFG043504 (1341)](http://webs.iiitd.edu.in/raghava/vactarbac/res.php?id=VFG043504) |
| Streptococcus pyogenes | [VFG000962  (1557)](http://webs.iiitd.edu.in/raghava/vactarbac/res.php?id=VFG000962) | [DEG10380037 (1380)](http://webs.iiitd.edu.in/raghava/vactarbac/res.php?id=DEG10380037) | [VFG005233 (1322)](http://webs.iiitd.edu.in/raghava/vactarbac/res.php?id=VFG005233) | [VFG000949 (1244)](http://webs.iiitd.edu.in/raghava/vactarbac/res.php?id=VFG000949) | [VFG000974 (1194)](http://webs.iiitd.edu.in/raghava/vactarbac/res.php?id=VFG000974) |
| Vibrio cholerae | [VFG000983  (5096)](http://webs.iiitd.edu.in/raghava/vactarbac/res.php?id=VFG000983) | [VFG002088 (2423)](http://webs.iiitd.edu.in/raghava/vactarbac/res.php?id=VFG002088) | [VFG000106 (2120)](http://webs.iiitd.edu.in/raghava/vactarbac/res.php?id=VFG000106) | [VFG007023 (1964)](http://webs.iiitd.edu.in/raghava/vactarbac/res.php?id=VFG007023) | [VFG002091 (1502)](http://webs.iiitd.edu.in/raghava/vactarbac/res.php?id=VFG002091) |

Note: * represent either VFDB or DEG ID, whereas ** represent the total number of predicted epitopes written inside the brackets.
